# Heterogeneity of Layer 1 Interneurons in the Mouse Medial Prefrontal Cortex

**DOI:** 10.1002/cne.70030

**Published:** 2025-03-04

**Authors:** Chen Shen, Wanpeng Cui, Wen‐Cheng Xiong, Lin Mei

**Affiliations:** ^1^ Department of Neurosciences, School of Medicine Case Western Reserve University Cleveland Ohio USA; ^2^ Louis Stokes Cleveland Veterans Affairs Medical Center Cleveland Ohio USA; ^3^ Chinese Institutes for Medical Research Beijing China; ^4^ Chinese Institute for Brain Research Beijing China; ^5^ Capital Medical University Beijing China

**Keywords:** electrophysiology, GABAergic inhibition, Layer 1 interneurons (L1INs), medial prefrontal cortex (mPFC), neurogliaform cells (NGCs), neuron‐derived neurotrophic factor (NDNF), optogenetics

## Abstract

Cortical Layer 1 (L1) acts as a critical relay for processing long‐range inputs. GABAergic inhibitory interneurons (INs) in this layer (Layer 1 interneurons [L1INs]) function as inhibitory gates, regulating these inputs and modulating the activity of deeper cortical layers. However, their characteristics and circuits in the medial prefrontal cortex (mPFC) remain poorly understood. Using biocytin labeling, we identified three distinct morphological types of mPFC L1INs: neurogliaform cells (NGCs), elongated NGCs (eNGCs), and single‐bouquet cell‐like (SBC‐like) cells. Whole‐cell recordings revealed distinct firing patterns across these subtypes: NGCs and eNGCs predominantly exhibited late‐spiking (LS) patterns, and SBC‐like cells displayed a higher prevalence of non‐LS (NLS) patterns. We observed both electrical and chemical connections among mPFC L1INs. Optogenetic activation of NDNF^+^ L1INs demonstrated broad inhibitory effects on deeper layer neurons. The strength of inhibition on pyramidal neurons (PyNs) and INs displayed layer‐specific preference. These findings highlight the functional diversity of L1INs in modulating mPFC circuits and suggest their potential role in supporting higher order cognitive functions.

## Graphical abstract

Layer 1 interneurons (L1INs) in the mouse medial prefrontal cortex (mPFC) exhibit extensive variety in morphology, firing pattern, and microcircuit function. On the basis of morphology, they could be classified into at least three subtypes—neurogliaform cells (NGCs), elongated NGCs (eNGCs), and single‐bouquet‐cell‐like cells (SBC‐like) that show distinct electrophysiological properties. Using double patch‐clamp recordings and optogenetic activations, we identified three key microcircuits involving L1INs: electrical coupling, lateral inhibition, and deeper layer inhibition, underscoring their critical roles in regulating prefrontal cortical circuits.

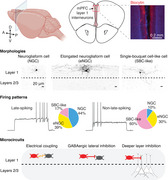

## 1 Introduction

The cerebral cortex plays a critical role in cognitive functions. In mammals, the neocortex typically consists of six layers, with Layer 1 (L1) being the most superficial (Douglas and Martin 2004). L1 lacks the cell bodies of pyramidal excitatory neurons (PyNs) (Ibrahim et al. 2020) but is populated with apical dendrites from neurons in deeper layers and axonal fibers originating from various subcortical regions (Cauller 1995; Cauller and Connors 1994; Petreanu et al. 2009; Schuman et al. 2021). This topological arrangement enables L1 to relay and process long‐range inputs, thereby modulating the activity of neurons in deep cortical layers. These functions are believed to be facilitated by a unique population of GABAergic interneurons (IN) in L1 (Layer 1 interneurons [L1INs]) (Letzkus et al. 2011; Muralidhar et al. 2013; Schuman et al. 2019; Tremblay et al. 2016). On the basis of morphology, L1INs can be grouped into three subtypes: neurogliaform cells (NGCs), elongated NGCs (eNGCs), and single‐bouquet‐cell‐like (SBC‐like) cells, whereas in terms of electrophysiological properties, L1INs display two main firing patterns in response to current injections: late‐spiking (LS) and non‐LS (NLS). The proportion of different L1IN subtypes varies from one cortex to another. For example, SBC‐like cells make up 66% of L1INs in the primary somatosensory cortex (S1) and 17% in the medial entorhinal cortex (MEC) (Schuman et al. 2019; Shi et al. 2023). Functionally, L1INs in the auditory cortex modulate learning‐related responses via dendritic inhibition (Abs et al. 2018), whereas L1INs in the somatosensory cortex contribute to the timing of sensory‐evoked responses through lateral inhibition (Fan et al. 2020). In the visual cortex, L1INs adjust their sensory responses according to different arousal states (Cohen‐Kashi Malina et al. 2021).

The medial prefrontal cortex (mPFC) plays a pivotal role in various cognitive processes, including attention, learning, working memory, and decision making (Coley et al. 2021; Euston et al. 2012; Menon and D'Esposito 2022; Xu et al. 2019). The mouse mPFC consists of the dorsal part of anterior cingulate area (ACAd), prelimbic area (PL), the medial part of the orbital area (ORBm), and infralimbic area (ILA) (Bakker et al. 2015; Carlén 2017; Lein et al. 2007; Wang et al. 2020). PyNs in the mouse mPFC form both intracortical connections and reciprocal long‐range projections to other brain regions (Anastasiades and Carter 2021; Brown and Hestrin 2009; Carmichael and Price 1996; Wang et al. 2006). They integrate inputs from multiple brain regions and exert top‐down cognitive control of cortical and subcortical structures (Mechelli et al. 2004; Miller and Cohen 2001; Ochsner et al. 2009; Rossi et al. 2009). Despite the critical role of mPFC in cognitive functions, its L1INs have not been carefully characterized.

Here, we studied mPFC L1INs using a variety of techniques. We characterized the morphology of mPFC L1INs via biocytin labeling and analyzed their electrophysiological properties through whole‐cell patch‐clamp recordings. Our results indicate that mPFC L1INs exhibit morphological subtypes and firing patterns comparable to those in the visual cortex, somatosensory cortex, and MEC (Jiang et al. 2015; Schuman et al. 2019; Shi et al. 2023). However, the proportions of these subtypes differ significantly (Tables 1 and 2), suggesting that mPFC L1INs may have different connectivity patterns with distinct functions. Moreover, our double whole‐cell patch‐clamp recording studies showed that mPFC L1INs form both electrical and chemical connections among themselves. By combining optogenetic activation with whole‐cell patch‐clamp recording from downstream neurons, we found that mPFC L1INs provide broad GABAergic inhibition of neurons in deeper cortical layers, with minimal inhibitory effects on L5/6 INs. These findings underscore an extensive inhibitory influence of L1INs within mPFC circuits and highlight the diverse connectivity and functions of L1INs.

**TABLE 1:** Morphological types of Layer 1 interneurons (L1INs) in different cortical areas.

	NGCs	eNGCs	SBC‐like cells	Ref.
mPFC	41.0% (16/39)	28.2% (11/39)	30.8% (12/39)	
MEC	57.7% (101/175)	25.7% (45/175)	16.6% (29/175)	Shi et al. (2023)
V1		34% (*n* = 84)	66%	Jiang et al. (2015)
S1		70%	30%	Schuman et al. (2019)

*Note:* Statistical data for MEC, V1, and S1 were obtained from their respective references. The proportion data for morphological subtypes in MEC were calculated from Figure S4B of Shi et al. (2023). Data for V1 were sourced from Figure 1E and the supplementary text on Layer 1 interneurons in Jiang et al. (2015). Data for S1 were derived from Figure 2G and Table 2 of Schuman et al. (2019). NGCs were not distinguished from eNGCs in Jiang et al. (2015) and Schuman et al. (2019). A chi‐squared test revealed statistically significant differences in the distribution of morphological types across brain regions (*χ* ^2^(df = 6) = 296.6, ****p < 0.0001, Cohen's w = 0.7266).

Abbreviations: eNGCs, elongated neurogliaform cells; MEC, medial entorhinal cortex; NGCs, neurogliaform cells; S1, primary somatosensory cortex; SBC‐like, single‐bouquet cell‐like; V1, primary visual cortex.

**TABLE 2:** Firing patterns of Layer 1 interneurons (L1INs) in different cortical areas.

	LS	NLS	BS	Ref.
mPFC	64.4% (67/104)	35.6% (37/104)		
MEC	76.0% (133/175)	24.0% (42/175)		Shi et al. (2023)
V1	32.7%	39.2%	28.1%	Jiang et al. (2015)
S1	37.4%	62.6%		Schuman et al. (2019)
Developing somatosensory cortex	76.4% (162/212) 79.7% (374/469)		23.6% (50/212) 20.3% (95/469)	Ma et al. (2014), Yao et al. (2016)

*Note:* Statistical data for MEC, V1, S1, and the developing somatosensory cortex were obtained from their respective references. The proportion data for firing patterns in MEC were calculated from Figure S4B of Shi et al. (2023). Data for V1 were derived by integrating Table S1 with Figure 1E from Jiang et al. (2015). Data for S1 were calculated by combining Figure 1B and Figure 2G from Schuman et al. (2019). Data for the developing somatosensory cortex were obtained from the results described in Ma et al. (2014) and Figure 2b by Yao et al. (2016). In Jiang et al. (2015), BS cells were distinguished from no‐burst NLS cells. However, we rarely observed BS cells in mPFC (1.1%, *n* = 2 out of 178 cells), and they were excluded from analysis due to the small sample size. A chi‐squared test revealed statistically significant differences in firing patterns across brain regions (*χ* ^2^(df = 10) = 451.8, ****p < 0.0001, Cohen's w = 0.6241).

Abbreviations: BS, burst spiking; LS, late‐spiking; MEC, medial entorhinal cortex; mPFC, medial prefrontal cortex; NLS, non‐late‐spiking; S1, primary somatosensory cortex; V1, primary visual cortex.

## 2 Materials and Methods

### 2.1 Mice

Experiments were approved by the Institutional Animal Care and Use Committee (IACUC) of Case Western Reserve University (RRID: SCR_011139). NDNF‐Cre mice (RRID: IMSR_JAX:030757) and wild‐type C57BL/6J mice (RRID: IMSR_JAX:000664) were obtained from the Jackson Laboratory. Mice were housed in the Case Western Reserve University animal resource center at room temperature with a 12‐h light/dark cycle and ad libitum access to food and water. Male mice aged 2–6 months were utilized.

### 2.2 Slice Preparation

Slice preparation and whole‐cell patch‐clamp recordings were performed as previously described (Cui et al. 2021; Pan et al. 2019). Briefly, mice were anesthetized with isoflurane and subjected to cardiac perfusion with ice‐cold, oxygenated slicing solution (110 mM choline chloride, 2.5 mM KCl, 0.5 mM CaCl_2_, 7 mM MgCl_2_, 1.3 mM NaH_2_PO_4_, 25 mM NaHCO_3_, and 20 mM d‐glucose, pH 7.3–7.4, 300–310 mOsm kg^−1^, saturated with 95% O_2_ and 5% CO_2_). After decapitation, the brain was rapidly removed, glued to a stage, and placed in ice‐cold oxygenated slicing solution. Coronal brain slices (300 µm thick) were cut using a vibratome (RRID: SCR_020243; VT1200S, Leica, Germany) and incubated in oxygenated recording solution at ∼34°C for 34 min. Slices were allowed to recover at room temperature for at least 30 min before recording. The solution used for incubation, recovery, and recordings consisted of 125 mM NaCl, 2.5 mM KCl, 2 mM CaCl_2_, 1.3 mM MgCl_2_, 1.3 mM NaH_2_PO_4_, 25 mM NaHCO_3_, and 10 mM d‐glucose (pH 7.3–7.4, 300–310 mOsm kg^−1^).

### 2.3 Electrophysiology Recordings

For whole‐cell patch‐clamp recordings, mPFC slices were transferred to a recording chamber and continuously perfused with oxygenated recording solution (3.0 mL min^−1^) at ∼31.7°C. Slices were visualized using infrared video microscopy and differential interference contrast optics (BX51WI, Olympus). Patch electrodes were fashioned from borosilicate glass capillaries (BF150‐86‐10, Sutter Instruments) with resistance ranging from 3 to 5 MΩ after being filled with a potassium‐based internal solution containing 125 mM K‐gluconate, 5 mM KCl, 10 mM HEPES, 10 mM Na_2_‐phosphocreatine, 0.2 mM EGTA, 4 mM Na_2_ATP, 0.3 mM Na_3_GTP, and 4 mM MgCl_2_ (pH 7.3–7.4, 285–290 mOsm kg^−1^). For morphological staining, 0.2% biocytin (Sigma) was added to the internal solution.

mPFC L1INs were identified and recorded on the basis of the location of their somata and their ability to generate action potentials. Other interneurons and pyramidal neurons (PyNs) were recorded and classified according to their location, morphology, membrane capacitance, and firing properties. Due to potential recording damage from oscillations in series resistance compensation, series resistance was not compensated but kept below 30 MΩ during recordings. Data were excluded if series resistance varied by more than 20%. Recording signals were amplified using an Axon 700B patch‐clamp amplifier, digitized with an Axon Digidata 1550B, filtered at 2.8 kHz, and sampled at 10 kHz. Analyses were performed using Clampex 10.7 (RRID: SCR_011323; Molecular Devices LLC) and MATLAB R2022a (RRID: SCR_001622; MathWorks, Natick, MA).

Intrinsic properties of L1INs were measured under current‐clamp conditions. Resting membrane potential (*V*_m_) was recorded immediately upon break‐in. Input resistance was calculated using Ohm's law from steady‐state voltage responses to −20 pA, 0.5 s current pulses. Membrane time constant was determined as the time taken for the potential to fall from its resting value to 63% (1 − 1/e) of its final value in response to −20 pA, 0.5 s current pulses. Cell capacitance was calculated using the following formula: c=r/rττ, where c is cell capacitance, r is input resistance, and τ is membrane time constant. Action potential characteristics were measured from action potentials evoked by 2 s just‐suprathreshold current pulses, initiated from a membrane potential near −70 mV. Spike threshold was determined by finding the potential at which the second derivative of the voltage waveform exceeded three times its standard deviation in the period preceding spike onset (Erisir et al. 1999). Spike peak amplitude was calculated as the difference between the peak and the threshold. Spike half‐width was measured at half the spike amplitude. Maximum spike rise slope was determined by the maximum value of the first derivative of the voltage waveform. Depolarizing hump amplitude was defined as the difference between the maximum potential within 200 ms after the onset of a just‐below‐threshold current step and the average potential over the final 300 ms of the same step (Schuman et al. 2019). First spike latency was timed from the onset of the just‐suprathreshold current injection to the initiation (threshold) of the first spike. Afterdepolarization (AHP) amplitude following bursts of spikes was measured as the difference in voltage between the steady‐state voltage and the minimum potential within 400 ms after the end of the +300 pA current injection. AHP duration was calculated as the time from the end of the +300 pA current injection until the membrane potential returned to a steady state.

For double whole‐cell patch‐clamp recordings, two L1INs within the same brain slice were simultaneously patched at their somata, which were located less than 200 µm apart. To test for electrical or chemical connections, repeated action potentials (APs) were induced in one cell using 600 pA, 2 ms current pulses at 5‐s intervals, whereas responses in the second cell were recorded either in current‐clamp mode (without current injection) or in voltage‐clamp mode (holding potential at +6 mV), respectively. To verify electrical coupling, 2‐s hyperpolarizing and depolarizing currents were injected into one cell, with voltage responses recorded in both cells. The strength of electrical coupling was quantified as the ratio of the membrane potential change in the non‐stimulated neuron to the change in the stimulated neuron during current injection. To assess chemical connections, bicuculline (10 µM) was introduced to inhibit GABAergic transmission upon detection of AP‐induced unitary inhibitory postsynaptic current (uIPSC).

### 2.4 Clustering Methodology

**FIGURE 1:**
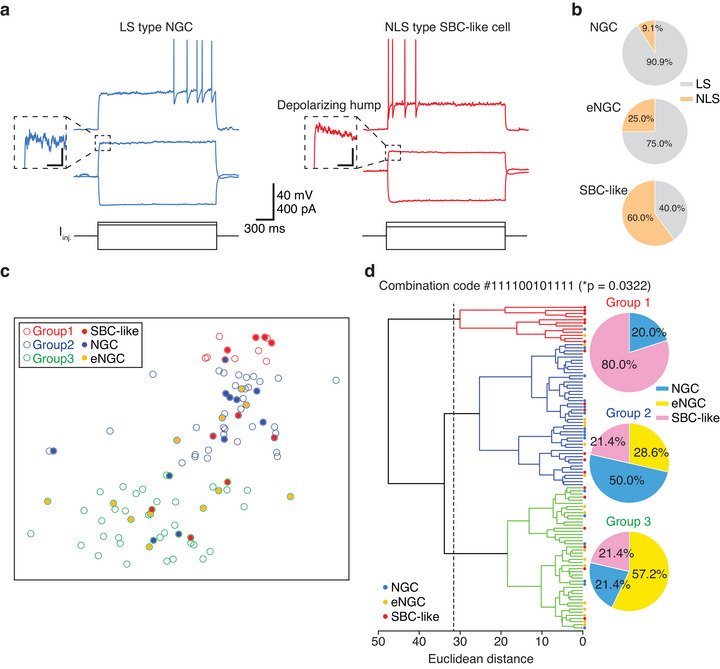
**Electrophysiological properties of morphologically distinct L1IN populations in the mPFC**. (a) Representative voltage traces from mPFC L1 late‐spiking (LS) type NGC (blue) and non‐late‐spiking (NLS) type SBC‐like (red) cells in response to hyperpolarizing, below‐threshold, and just‐suprathreshold current injections (*I_inj._*, black). (b) Pie charts illustrate the proportions of firing patterns within each morphological subgroup (NGC, LS = 10, NLS = 1; eNGC, LS = 9, NLS = 3; SBC‐like, LS = 4, NLS = 6; *χ* ^2^(df = 2) = 6.679, *p = 0.0355, Cohen's w = 0.4499). (c) A t‐SNE projection of 104 recorded L1INs based on the nine electrophysiological parameters used in (d). (d) Dendrogram of nine electrophysiological parameters. *Note:* See also Table 4. (a) LS firing patterns were predominantly observed in NGCs and eNGCs, whereas NLS firing patterns were mostly seen in SBC‐like cells. The current steps are shown in black at the bottom. Scale bars, 40 mV or 400 pA × 300 ms. Insets show zoomed‐in images from the onset of below‐threshold current injections, highlighting the depolarizing hump observed in non‐late‐spiking cells. Scale bars, 2 mV × 200 ms. (c) Each circle represents a single cell. Outline color indicates the electrophysiological group (red: Group 1, blue: Group 2, green: Group 3) assigned by hierarchical clustering, whereas the fill color corresponds to morphological identity (red: SBC‐like, blue: NGC, yellow: eNGC). (d) A hierarchical cluster tree was constructed using Ward's method (see Section 2.4), revealing three distinct electrophysiological cell groups. The parameters used for grouping were resting membrane potential, threshold, peak amplitude, spike half‐width, AHP amplitude, input resistance, membrane time constant, cell capacitance, and depolarizing hump (see Figure S2a). Colored dots between the dendrogram and the pie charts indicate morphological identity (blue: NGC, yellow: eNGC, red: SBC‐like), as shown in (c). The pie charts show the proportions of morphological types within each group (Group 1, NGC = 1, eNGC = 0, SBC‐like = 4; Group 2, NGC = 7, eNGC = 4, SBC‐like = 3; Group 3, NGC = 3, eNGC = 8, SBC‐like = 3). A chi‐squared test (*χ* ^2^(df = 4) = 10.55, *p = 0.0322, Cohen's w = 0.5654) indicates a statistically significant association between morphological type and these electrophysiological groupings.

Electrophysiological property–dependent cluster analysis was conducted as previously described with modifications (Schuman et al. 2019; Shi et al. 2023). A total of 104 mPFC L1INs were analyzed for 12 intrinsic electrophysiological properties: resting membrane potential (mV), spike threshold (mV), spike peak amplitude (mV), spike half‐width (ms), first spike latency (ms), spike maximum rise slope (mV/ms), afterhyperpolarization (AHP) amplitude (mV), AHP duration (ms), input resistance (MΩ), membrane time constant (ms), cell capacitance (pF), and depolarizing hump (mV) (see Section 2.3). L1INs were grouped by Ward's hierarchical clustering analysis with threshold Euclidean distances set to ensure the formation of three distinct clusters. Associations between the clusters and three morphological subtypes were determined by screening all possible non‐empty subsets of the 12 intrinsic properties (4095 or 2^12^ − 1 in total) (Figure S2a) and subjected to chi‐squared tests. A total of 287 subsets produced clusters that were significantly associated with morphological subtypes. After weighing each electrophysiological property in the significant subsets, we identified a subset containing the top 9 parameters that fully covered 199 significant subsets (∼69% of 287). The significance of the association between clusters and morphology was lost if a 10th parameter was included. The binary code for the nine‐parameter subset was #111100101111, corresponding to the following properties: resting membrane potential (mV), spike threshold (mV), spike peak amplitude (mV), spike half‐width (ms), AHP amplitude (mV), input resistance (MΩ), membrane time constant (ms), cell capacitance (pF), and depolarizing hump (mV). This nine‐parameter subset was utilized for subsequent correlation analyses of electrophysiological clustering and morphologies (Figure 1c,d).

To cross‐validate the effectiveness of Ward's hierarchical clustering, we determined whether randomly shuffled electrophysiological parameters could generate clusters significantly associated with morphological subtypes in a permutation test. Of the 4095 non‐empty subsets derived from the shuffled data, none exceeded the significance threshold (Figure S2b). These results demonstrate that the observed associations between electrophysiological clusters and morphologies are unlikely to have arisen by chance.

Additionally, to confirm the appropriateness of the 9‐parameter subset for correlating electrophysiological clustering with morphologies, we performed 1000 shuffles of each electrophysiological parameter across cells and evaluated the correlation with morphologies. Only 21 out of 1000 shuffles (≈2.1%) achieved a *p* value below 0.05, which is below the conventional 5% significance level (Figure S2c). These findings demonstrate the validity of our clustering methodology and the selection of the nine‐parameter subset.

### 2.5 Immunohistochemistry and Morphological Reconstructions

Morphological recovery and fluorescent microscopy examination were conducted as described previously with modifications (Gouwens et al. 2020; Jiang et al. 2015; Pan et al. 2019; Sun et al. 2021). After whole‐cell patch‐clamp recordings, brain slices were fixed by immersion in freshly prepared 4% paraformaldehyde (PFA) in 0.1 M phosphate‐buffered saline (PBS, pH 7.4) at 4°C for at least 48 h. The fixed brain slices were then washed in 0.1 M PBS for 10 min to remove PFA residues and subsequently blocked with 10% donkey serum at room temperature for 2 h. This was followed by permeabilization with a high concentration of detergents (4% Triton X‐100 in PBS) at room temperature for 2 h. Slices were then incubated in PBS containing AlexaFluor‐488 streptavidin conjugate (Thermo Fisher Scientific RRID: SCR_008452; S11223, diluted 1:500) or AlexaFluor‐647 streptavidin conjugate (RRID: AB_2336066; Thermo Fisher Scientific, S21374, diluted 1:500), and 4% Triton X‐100 for 2 days at 4°C. After streptavidin staining, slices were counterstained with DAPI for 15 min and mounted with an aqueous mounting solution (Aqua‐Mount, Lerner Laboratories, Thermo Fisher Scientific) for fluorescent microscopy.

In some experiments, slices were stained for GABA after permeabilization, prior to streptavidin staining. In brief, brain slices were incubated in a blocking solution with rabbit anti‐GABA antibodies (RRID: AB_477652; Sigma‐Aldrich, A2052, diluted 1:500) for 2 days at 4°C. Afterward, sections were washed with 0.2% Triton X‐100 for at least 1 h before overnight incubation with goat anti‐rabbit IgG conjugated to AlexaFluor‐594 (RRID: AB_143165; Thermo Fisher Scientific, A11008, diluted 1:1000 in blocking solution) at 4°C. Slices were washed with 0.2% Triton X‐100 for 15 min before proceeding to streptavidin staining.

Morphologically recovered cells were examined, reconstructed, and analyzed using a 63X oil‐immersion objective lens on the ZEISS LSM 880 system. Z‐series images were captured in 1–3 µm increments and subsequently processed and analyzed with ImageJ software and custom MATLAB code.

### 2.6 Surgical Procedures and Intracranial Injection

NDNF‐Cre mice were used for optogenetic studies of mPFC L1INs. Mice were anesthetized with isoflurane and secured in a stereotaxic frame (David Kopf Instruments). Isoflurane was delivered in 21% oxygen (Wilding et al. 2017) at a rate of 500–700 mL min^−1^, with the concentration adjusted throughout the procedure (induction: 3%–4% [vol vol^−1^], surgery: 0.9%–1.5% [vol vol^−1^]) to maintain stable breathing and anesthesia depth. Anesthesia depth was assessed via the toe‐pinch reflex. Ophthalmic ointment (Systane) was applied for eye protection, and the fur on the head area was shaved before surgery. After exposing the skull, head‐holding angles were adjusted using a stereotaxic alignment indicator (David Kopf Instruments) to ensure the skull was leveled (bregma and lambda at equal heights) and aligned with the stereotaxic frame axes. Body temperature was maintained at 37°C using a heating pad throughout surgery.

As described previously (Gao et al. 2023; Wang et al. 2023), stereotaxic injections were performed using glass pipettes (Cat# 5‐000‐1001‐X10, Drummond) pulled with a micropipette puller (RRID: SCR_021042; P1000, Sutter, USA) to achieve outer diameters of 30–50 µm. Glass pipettes were polished at the tips (Micro Forge MF‐830, Narishige, Japan) and mounted on a microinjection pump (Nanoliter 2010 Injector, WPI). AAV‐EF1a‐DIO‐hChR2(H134R)‐EYFP (UNC Vector Core; RRID: SCR_023280) was injected bilaterally at a rate of 50–100 nL min^−1^, with a volume of 300 nL and a titer of 1 × 10^12^ vg mL^−1^. Injection sites were determined using the following coordinates: mPFC [anterior–posterior (AP) = +2.10 mm, medial–lateral (ML) = ±0.25 mm, and dorsal–ventral (DV) = from −1.40 mm retracted to −1.20 mm during viral infusion].

### 2.7 Optogenetic Stimulation

Optogenetic stimulation during slice recordings was performed as previously described (Liu et al. 2022; Zou et al. 2020). To activate mPFC L1INs, 300 nL of AAV‐EF1a‐DIO‐ChR2‐EYFP was bilaterally injected into the mPFC of adult NDNF‐Cre mice. After a recovery period of at least 3 weeks for viral expression, whole‐cell patch‐clamp recordings were performed as described above using the potassium‐based internal solution. Laser pulses (473 nm, 5 ms) were generated by a DPSS laser (Ultralasers Inc.) and delivered via an optic fiber (Thorlabs, # RJPFF2) to stimulate ChR2‐expressing neurons.

Light‐evoked APs from NDNF^+^ L1INs were recorded in current‐clamp mode, whereas deep‐layer neurons were recorded for light‐evoked currents in voltage‐clamp at a holding potential of +6 mV (Cruikshank et al. 2012). Bicuculline (10 µM) was added after detecting IPSCs to validate GABAergic transmission. To confirm monosynaptic transmission, TTX (1 µM) and 4‐AP (200 µM) were sequentially applied to the recording solution to block polysynaptic transmission (Linders et al. 2022; Wang et al. 2022).

### 2.8 Quantification and Statistical Analysis

**FIGURE 2:**
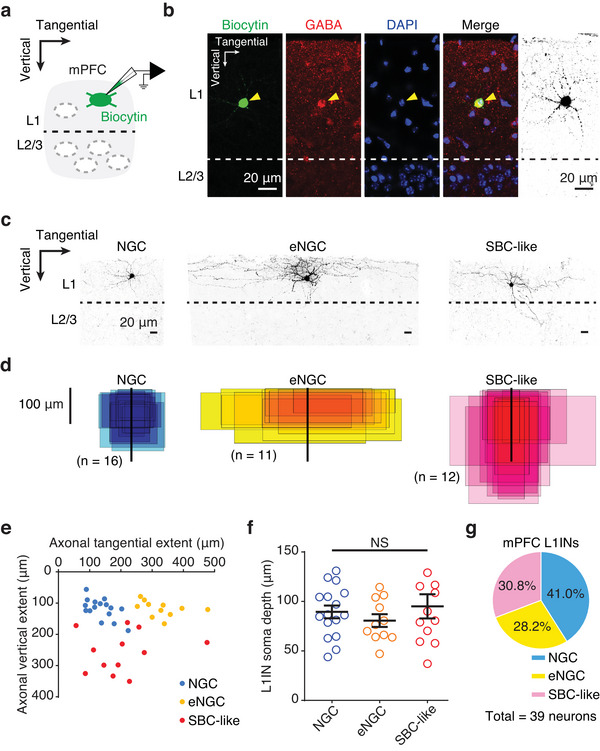
**Morphologically distinct L1IN populations in the mPFC**. (a) Cartoon illustrating a whole‐cell patch‐clamp recording of an L1 cell in the mPFC, with biocytin infusion for post hoc morphological reconstruction. (b) Recorded neurons were co‐stained with GABA (red) and DAPI (blue) to ensure that GABAergic L1INs of the mPFC were used for analysis. (c) Three morphological types of mPFC L1INs were identified: neurogliaform cells (NGC), elongated NGCs (eNGC), and single‐bouquet cell‐like cells (SBC‐like). (d) Tangential and vertical extent of axonal projections from morphologically distinct mPFC L1INs. Scale bar, 100 µm. (e) Scatterplot showing the tangential and vertical extents of axonal projections for the three L1IN populations corresponding to (d). (f) Summary data on the soma depth of recorded L1INs (NGC, *n* = 16 cells; eNGC, *n* = 11 cells; SBC‐like, *n* = 12 cells; one‐way ANOVA, F(2, 36) = 0.6375, *p* = 0.5345, *η* ^2^ = 0.0342). (g) Pie chart showing the proportions of the three morphological types. The bar graphs depict the mean ± SEM. *Note:* See also Table 3. (a) Arrows indicate the vertical and tangential axes of the medial wall of the prefrontal cortex. (b) Scale bars, 20 µm. (c) Scale bars, 20 µm. (d) The axonal extents of NGC (*n* = 16 cells) are shown in blue, eNGC (*n* = 11 cells) in yellow, and SBC‐like (*n* = 12 cells) in pink. The somata of each morphological group are aligned along the same vertical axis, with color intensity increasing with the degree of overlap.

Results were reported as mean ± SEM. Data were analyzed with GraphPad Prism 9 (RRID: SCR_002798), Microsoft Excel (RRID: SCR_016137), ImageJ (RRID: SCR_003070), MATLAB R2022a (RRID: SCR_001622), and Clampex 10.7 (RRID: SCR_011323). Chi‐squared tests were conducted to assess whether the morphological subtypes were evenly distributed (Figure 2g) and to determine if the morphological subtypes were associated with firing patterns (Figure 1b) or electrophysiological groupings (Figure 1d). Two‐tailed paired or unpaired Student's *t*‐tests were employed where appropriate. For multiple group comparisons, one‐way ANOVA followed by Tukey's post hoc multiple comparison test was used. Simple linear regression was performed to assess correlations between variables. Repeated measures (RM) ANOVA was used when the same subjects were tested across groups. If missing values prevented one‐way RM ANOVA, a restricted maximum likelihood (REML) mixed‐effects model was used instead. Statistical significance was set at *p < 0.05. Detailed information is provided in the figure legends.

## 3 Results

### 3.1 mPFC L1INs With Different Morphological Features

L1, characterized by its sparse cellular density, was identified by a line parallel to the pia, below which were Layer 2/3 (L2/3) with a sharp increase in neuronal somata (Baillarger 1840; Wagener et al. 2015). To characterize the morphology and electrophysiological properties of mPFC L1INs, slices were prepared from adult mice, and cells in L1 were recorded using whole‐cell patch‐clamp techniques with pipettes filled with biocytin (Figure 2a and Figure S1a,b). After recordings, slices were stained with fluorescent conjugates of streptavidin to visualize biocytin and with anti‐GABA antibodies to identify interneurons. Recorded cells were considered L1INs based on their somatic location, ability to generate action potentials, and GABA expression (Figure 2b). Most reconstructed L1INs (26 out of 39 cells) were located in the prelimbic area, L1 (PL1), per a standard atlas template (Bakker et al. 2015; Lein et al. 2007), as shown in Figure S1c. In comparison, the other 13 cells were distributed across the anterior cingulate area, dorsal part, L1 (ACAd1); the orbital area, medial part, L1 (ORBm1); and the infralimbic area, L1 (ILA1) (Figure S1d).

Of the 39 morphologically reconstructed L1INs, 3 distinct populations were identified on the basis of biocytin‐staining patterns: 41.0% were NGCs (*n* = 16 cells), 28.2% were eNGCs (*n* = 11 cells), which were distinguished from classic NGCs by their extended axonal arborizations, and 30.8% were single‐bouquet cell‐like (SBC‐like) cells (*n* = 12 cells) with side branches extending to deeper cortical layers (Figure 2c,d). These morphological subtypes were distinct from one another in their exonal extensions. Quantitative analysis showed that NGCs typically occupied a cubic area with sides of approximately 100 µm (Figure 2d,e). In contrast, eNGCs displayed the longest tangential extents (NGC = 137.2 ± 10.6 µm; eNGC = 328.4 ± 19.8 µm; SBC‐like = 202.3 ± 31.5 µm; one‐way ANOVA, F(2, 36) = 21.43, ****p < 0.0001, and an effect size *η* ^2^ = 0.5435), whereas SBC‐like cells exhibited the greatest vertical extents (NGC = 118.2 ± 8.2 µm; eNGC = 118.0 ± 7.5 µm; SBC‐like = 256.9 ± 18.9 µm; one‐way ANOVA, F(2, 36) = 41.43, ****p < 0.0001, *η* ^2^ = 0.6971) (Figure 2d,e). These morphological traits are consistent with findings from other cortical areas, such as the S1 (Schuman et al. 2019) and the MEC (Shi et al. 2023), indicating conserved L1IN subtypes across cortical regions.

Previous studies have shown that L1 can be divided into two sublayers in the S1 and the MEC, with SBC‐like cells typically residing in the lower half of L1 (Schuman et al. 2019; Shi et al. 2023). To determine whether the morphological subtypes of mPFC L1INs are distributed at different depths, we analyzed the distance from their soma to the pia mater among the three morphological groups. However, no significant differences in soma depth were observed (NGC = 89.5 ± 6.4 µm; eNGC = 80.7 ± 6.4 µm; SBC‐like = 95.1 ± 12.3 µm; one‐way ANOVA, F(2, 36) = 0.6375, *p* = 0.5345, *η* ^2^ = 0.0342) (Figure 2f), suggesting a uniform soma distribution across L1IN morphological subtypes in the mPFC. Additionally, the proportions of the three morphological subtypes were relatively balanced (NGC = 41.0%; eNGC = 28.2%; SBC‐like = 30.8%) (Figure 2g). To test whether the morphological subtypes of L1INs were evenly distributed, we performed Pearson's chi‐squared goodness‐of‐fit test. The analysis yielded a *χ* ^2^(df = 2) = 0.5170, *p* = 0.7722, and an effect size of Cohen's w = 0.1151. These results indicate that the population of L1INs in the mPFC is evenly distributed, suggesting that all three L1IN types may have distinct yet equally important roles in mPFC function.

### 3.2 Firing Patterns of L1INs

To characterize the electrophysiological properties of mPFC L1INs, we recorded and measured 12 intrinsic properties from L1INs in adult male mice under current‐clamp conditions. These properties included cell capacitance, membrane time constant, input resistance, resting membrane potential, spike threshold, spike peak amplitude, spike half‐width, maximum spike rise slope, depolarizing hump, first spike latency, afterhyperpolarization (AHP) amplitude, and AHP duration (see Section 2.3). The dataset used for analysis comprised 104 mPFC L1INs, of which 33 were subsequently registered with their morphological features (NGCs, *n* = 11 cells; eNGCs, *n* = 12 cells; SBC‐like, *n* = 10 cells) and used to associate morphology with electrophysiological properties.

Cortical L1INs can be classified into LS or NLS firing patterns based on the time delay of action potential onset during just‐suprathreshold current injections (Chu et al. 2003; Jiang et al. 2013; Schuman et al. 2019) (Figure 1a). To explore whether firing patterns are associated with the morphological subtypes of L1INs, we examined the firing patterns within each subtype. We found that NGCs and eNGCs predominantly exhibited the LS firing patterns (NGC = 10 out of 11 cells, 90.9%; eNGC = 9 out of 12 cells, 75.0%), whereas SBC‐like cells mostly displayed NLS firing patterns (*n* = 6 out of 10 cells, 60.0%) (Figure 1a,b). A chi‐squared test of independence yielded *χ* ^2^(df = 2) = 6.679, *p = 0.0355, and an effect size of Cohen's w = 0.4499. These results indicate an association between morphological types and firing patterns, suggesting that certain morphological types are more likely to exhibit specific firing patterns (Figure 1b). However, no group exhibited a completely uniform firing pattern, suggesting that firing patterns alone cannot reliably define the morphological features of mPFC L1INs and vice versa.

To investigate whether any intrinsic properties are associated with morphological subtypes, we examined the 12 intrinsic properties within each subtype (Table 3 and Table S1). We found that SBC‐like cells exhibited significantly higher input resistance compared to eNGCs (SBC‐like = 539.2 ± 100.3 MΩ; eNGC = 299.5 ± 23.0 MΩ; Tukey's post hoc, *p = 0.0174), whereas cell capacitance was similar between the two groups (SBC‐like = 28.7 ± 1.8 pF; eNGC = 26.7 ± 1.3 pF; Tukey's post hoc, *p* = 0.8109). This suggests that the number of open ion channels may differ between SBC‐like cells and eNGCs. However, other intrinsic properties did not significantly differ across morphological subtypes (Table 3). These findings indicate that morphology and electrophysiological properties are not fully interdependent in L1INs.

**TABLE 3:** Comparison of intrinsic electrophysiological properties of medial prefrontal cortex (mPFC) Layer 1 interneurons across different morphological groups.

	NGC (*n* = 11)	eNGC (*n* = 12)	SBC‐like (*n* = 10)	*p* values
Cell capacitance (pF)	31.0 ± 3.2	26.7 ± 1.3	28.7 ± 1.8	0.3519, 0.7453, 0.8109
Time constant (ms)	13.4 ± 2.1	11.7 ± 1.4	18.5 ± 3.0	0.8436, 0.2556, 0.0893
Input resistance (MΩ)	372.1 ± 33.5	299.5 ± 23.0	539.2 ± 100.3	0.6391, 0.1302, 0.0174*
Resting *V*_m_ (mV)	−72.6 ± 2.1	−71.4 ± 2.1	−68.2 ± 1.3	0.8920, 0.2594, 0.4668
Threshold (mV)	−32.4 ± 1.5	−34.7 ± 1.1	−36.4 ± 2.4	0.5649, 0.2383, 0.7724
Peak amplitude (mV)	48.2 ± 3.1	53.1 ± 2.6	53.2 ± 5.2	0.6037, 0.6146, 0.9996
Half‐width (ms)	1.2 ± 0.1	1.2 ± 0.1	1.2 ± 0.1	0.9995, 0.9776, 0.9694
Max spike slope (mV/ms)	125.1 ± 12.4	135.5 ± 12.1	150.1 ± 20.0	0.8687, 0.4817, 0.7672
Depolarizing hump (mV)	0.9 ± 0.4	0.7 ± 0.3	1.3 ± 0.5	0.9411, 0.7529, 0.5459
First spike latency (ms)	1037.4 ± 166.6	734.4 ± 154.5	559.2 ± 161.1	0.3742, 0.1176, 0.7258
AHP amplitude (mV)	1.7 ± 0.3	2.4 ± 0.3	2.0 ± 0.5	0.3367, 0.7741, 0.7657
AHP duration (ms)	462.1 ± 101.5	527.1 ± 130.1	557.7 ± 118.1	0.9171, 0.8436, 0.9820

*Note:* Data are presented as mean ± SEM. For details on the computation of parameters, refer to Section 2.3. Statistical differences (*p* values) were assessed using ordinary one‐way ANOVA followed by Tukey's post hoc multiple comparison test. The reported *p* values represent comparisons between NGC versus eNGC, NGC versus SBC‐like, and eNGC versus SBC‐like, respectively. Effect sizes are reported in Table S1. *V*_m_ refers to membrane potential; AHP refers to afterdepolarization. Relates to Figure 2.

To determine whether L1INs can be grouped by electrophysiological properties, we performed unsupervised hierarchical cluster analysis using Ward's method on 4095 (2^12^ − 1) subsets generated from 12 intrinsic properties of 104 mPFC L1INs (Figure S2a–c; Section 2.4). Among these subsets, 287 showed a significant association between morphological types and electrophysiological groupings (Figure S2a). From these, we selected the subset containing 9 electrophysiological properties—resting membrane potential, threshold, peak amplitude, spike half‐width, AHP amplitude, input resistance, membrane time constant, cell capacitance, and depolarizing hump—that exhibited a significant association with morphological features. This subset was used to generate the t‐SNE projection (Figure 1c) and the dendrogram of electrophysiological groupings (Figure 1d). A chi‐squared test revealed a significant association between morphological types and electrophysiological groups (*χ* ^2^(df = 4) = 10.55, *p = 0.0322, Cohen's w = 0.5654) (Figure 1d). In this grouping, SBC‐like cells comprised 80.0% of the morphologically registered cells in Group 1 (NGC = 1, eNGC = 0, SBC‐like = 4). NGCs made up 50.0% of registered cells in Group 2 (NGC = 7, eNGC = 4, SBC‐like = 3), whereas eNGCs accounted for 57.2% of registered cells in Group 3 (NGC = 3, eNGC = 8, SBC‐like = 3). Examination of the 12 intrinsic properties within each electrophysiological group (Table 4) revealed significant differences in membrane time constant and input resistance. Additionally, the spike threshold differed significantly between Groups 2 and 3.

**TABLE 4:** Comparison of intrinsic electrophysiological properties of medial prefrontal cortex (mPFC) Layer 1 interneurons across groups.

	Group 1 (*n* = 12)	Group 2 (*n* = 46)	Group 3 (*n* = 46)	*p* values
Cell capacitance (pF)	30.7 ± 1.8	29.3 ± 1.2	25.9 ± 1.1	0.8487, 0.1382, 0.0876
Time constant (ms)	26.5 ± 1.3	15.2 ± 1.1	9.8 ± 0.4	<0.0001, <0.0001, <0.0001
Input resistance (MΩ)	809.8 ± 62.6	403.5 ± 11.2	234.6 ± 6.3	<0.0001, <0.0001, <0.0001
Resting *V*_m_ (mV)	−68.4 ± 1.7	−69.6 ± 1	−72.1 ± 0.8	0.8276, 0.1554, 0.1221
Threshold (mV)	−38.2 ± 1.8	−37.5 ± 0.8	−34.3 ± 0.7	0.9185, 0.0678, 0.0131*
Peak amplitude (mV)	50.4 ± 4	52.2 ± 1.4	55.1 ± 1.6	0.8638, 0.3733, 0.4038
Half‐width (ms)	1.2 ± 0.1	1.2 ± 0	1.3 ± 0.2	0.9973, 0.9704, 0.9650
Max spike slope (mV/ms)	128.6 ± 14.8	128.8 ± 5.5	149.2 ± 7.6	>0.9999, 0.3540, 0.0883
Depolarizing hump (mV)	1.2 ± 0.4	0.8 ± 0.2	1.3 ± 0.2	0.5719, 0.9936, 0.1961
First spike latency (ms)	465.1 ± 138.7	674.4 ± 75.6	674.3 ± 72	0.4009, 0.4011, >0.9999
AHP amplitude (mV)	1.4 ± 0.4	1.5 ± 0.1	1.9 ± 0.2	0.9541, 0.2580, 0.1161
AHP duration (ms)	524 ± 107.3	360.4 ± 33.9	531.1 ± 55.1	0.2550, 0.9973, 0.0303*

*Note:* Data are presented as mean ± SEM. For details on parameter computation, refer to Section 2.3. Statistical differences (*p* values) were assessed using ordinary one‐way ANOVA followed by Tukey's post hoc multiple comparison test. The reported *p* values represent comparisons between Group 1 versus Group 2, Group 1 versus Group 3, and Group 2 versus Group 3, respectively. Effect sizes are reported in Table S1. *V*_m_ refers to membrane potential; AHP refers to afterdepolarization. Relates to Figure 1d.

Besides the 9‐parameter subset, we tested the subset containing all 12 intrinsic properties (Figure S2d) and the subset comprising only action potential waveform measures (Figure S2e). However, neither subset exhibited a significant association between morphological features and electrophysiological groupings.

### 3.3 L1INs Communicate With Each Other Through Electrical or Chemical Connections

**FIGURE 3:**
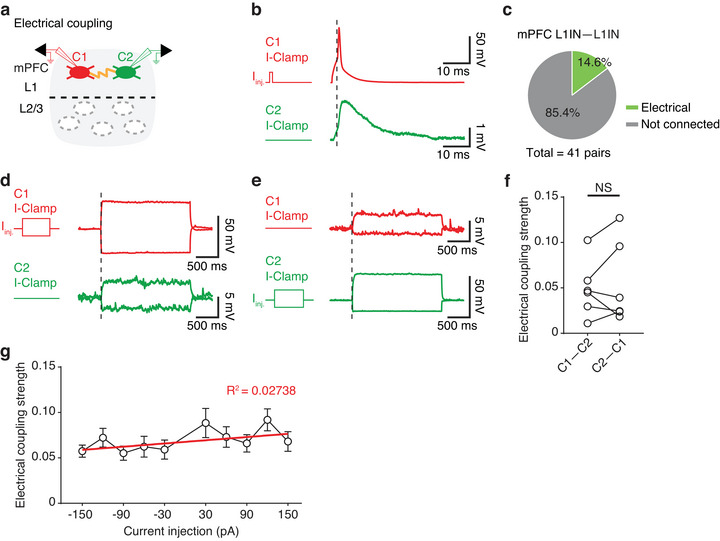
**Electrical connections between mPFC L1INs**. (a) Cartoon illustrating simultaneous whole‐cell recordings of two mPFC L1 cells (C1 in red, C2 in green) connected via electrical coupling. (b) An action potential elicited in C1 induced an immediate depolarizing response in C2 with no time delay. (c) Pie chart showing the proportion of electrically coupled pairs among all recorded pairs (electrical coupling, *n* = 6 out of 41 pairs). (d) Hyperpolarizing and depolarizing current injections in C1 elicited corresponding responses in C2. (e) Current injection in C2 induced corresponding responses in C1. (f) The electrical coupling strength between C1 and C2 was not significantly different in either direction (*n* = 6 pairs; paired *t*‐test, *p* = 0.5587, Cohen's *d* = 0.1612). (g) The electrical coupling strength was not correlated with the amount of current injected. *Note:* (b) The dashed line indicates the spike threshold of C1. The left graph shows the current injection (*I_inj._*) in C1 and the response of C2 recorded under current‐clamp conditions. Scale bars, 50 mV × 10 ms and 1 mV × 10 ms. (d) The dashed line indicates the onset of current injection in C1. Scale bars, 50 mV × 500 ms and 5 mV × 500 ms. (e) Scale bars, 5 mV × 500 ms and 50 mV × 500 ms.

To investigate the interconnectivity of L1INs in the mPFC, we conducted simultaneous double whole‐cell patch‐clamp recordings in brain slices from wildtype adult male mice. We selected pairs of L1INs with somata located less than 200 µm apart for recording (Figure 3a). To assess the electrical coupling between L1INs, we injected a depolarizing current into one neuron while recording the response from the other. The electrical coupling was identified when the depolarizing current produced an immediate depolarizing response in the non‐stimulated neuron (Figure 3b). This response persisted despite the bath application of glutamate receptor inhibitors (CNQX [20 µM] and DL‐AP5 [50 µM]) and a GABA receptor inhibitor (bicuculline methiodide [BMI, 10 µM]), confirming that the connection was electrical rather than chemical. Of the 41 recorded L1IN pairs, 6 pairs (14.6%) exhibited electrical coupling (Figure 3c).

To further validate electrical coupling, we delivered 2‐s subthreshold depolarizing or hyperpolarizing current injections to one neuron while monitoring voltage changes in the non‐stimulated neuron (Figure 3d). The voltage changes in the non‐stimulated neuron were independent of action potential events, indicating that the pairs were connected via electrical coupling rather than chemical connections. To assess whether the electrical coupling was bidirectional, we alternated current injections between the two neurons and observed similar voltage responses in the non‐stimulated neurons (Figure 3e). To analyze if the electrical coupling had a directional preference, we quantified the coupling strength in both directions (calculated as the voltage change in the non‐stimulated neuron divided by the voltage change in the stimulated neuron) and found no significant differences between directions (0.05 ± 0.01 vs. 0.05 ± 0.02; paired *t*‐test, *p* = 0.5587, Cohen's *d* = 0.1612) (Figure 3f), suggesting the electrical coupling has no directional preference. Further analysis revealed no correlation between electrical coupling strength and the amplitude of injected current (linear regression, *R* ^2^ = 0.02738) (Figure 3g). These findings demonstrate that electrical coupling among mPFC L1INs is a stable characteristic with an average coupling strength of 0.05 ± 0.01, independent of stimulation direction or intensity.

**FIGURE 4:**
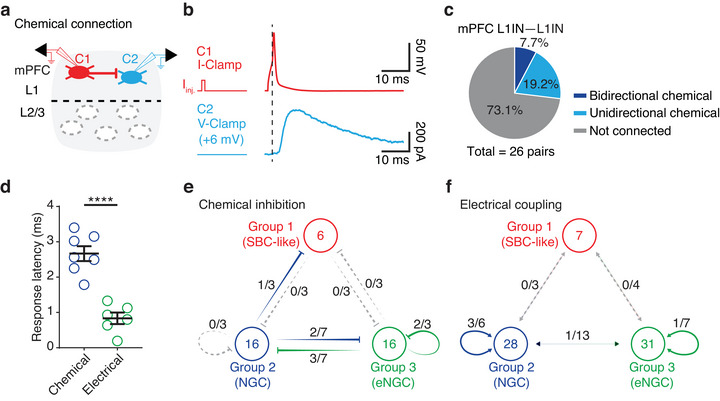
**Chemical connections between mPFC L1INs**. (a) Cartoon illustrating simultaneous whole‐cell recordings of two mPFC L1 cells (C1 in red, C2 in blue) connected by chemical synapses. (b) An action potential elicited in C1 induced an IPSC response in C2 with a time delay. (c) Pie chart showing the proportions of chemical connection pairs among all recorded pairs (bidirectional, *n* = 2 out of 26 pairs; unidirectional, *n* = 5 out of 26 pairs). (d) The response latency for electrical connections was significantly shorter than for chemical connections (chemical connection, *n* = 7 pairs; electrical connection, *n* = 6 pairs; unpaired *t*‐test, ****p < 0.0001, Cohen's *d* = 1.7258). (e) Chemical connections among mPFC L1IN groups. (f) Electrical connections among mPFC L1IN groups. *Note:* The bar graphs depict the mean ± SEM. (b) The dashed line indicates the spike threshold of C1. The left graph shows the current injection (*I_inj._*) in C1 under current‐clamp conditions, whereas C2 was recorded using a +6 mV voltage‐clamp. Scale bars, 50 mV × 10 ms and 200 pA × 10 ms. (e) Groups 1–3, as defined in (Figure 1d), are represented by circles. The numbers within each circle indicate the number of neurons recorded under double‐patch recording conditions. Lines terminating with a flat end denote the direction of chemical inhibition between groups. The fraction adjacent to each line represents the proportion of connected pairs out of the total recorded pairs. (f) Bidirectional electrical connections between groups are represented by lines with arrows.

In addition to electrical coupling, chemical connections between mPFC L1INs were identified through paired recordings (Figure 4a,b). To test for chemical connections, we injected a suprathreshold current into one neuron to elicit an action potential while recording the uIPSC in the non‐stimulated neuron (Figure 4b). These inhibitory responses were blocked by the bath application of BMI (10 µM), confirming that the connections were GABAergic. Of the 26 pairs of L1INs, 7 pairs (26.9%) displayed chemical connections, with 2 pairs (7.7%) showing bidirectional chemical connections and 5 pairs (19.2%) exhibiting unidirectional chemical connections (Figure 4c).

When comparing response latencies between electrical and chemical connections, we found that electrical connections had significantly shorter latencies than chemical connections (chemical connection = 2.6 ± 0.2 ms; electrical connection = 0.8 ± 0.2 ms; unpaired *t*‐test, ****p < 0.0001, Cohen's *d* = 1.7258) (Figure 4d). The shorter latency of electrical coupling suggests a role in the rapid synchronization of neuronal activities, whereas GABAergic inhibition likely differentiates the functions of various L1IN groups. By incorporating the double patch recorded neurons into the three distinct electrophysiological cell groups (as shown in Figure 1c,d), we constructed simplified connectivity maps. For chemical connections among mPFC L1IN groups (Figure 4e), we observed high connectivity between Groups 2 and 3, with five out of seven recorded pairs (71.4%) exhibiting chemical connections. Additionally, Group 3 demonstrated intra‐group chemical inhibition, with two out of three recorded pairs (66.7%) showing connections within the group. Regarding electrical connections (Figure 4f), there was substantial connectivity within Group 2, as three out of six pairs (50.0%) were electrically coupled. However, only six to seven neurons were classified into Group 1 under double patch recordings, limiting our ability to draw definitive conclusions about its connectivity patterns. Overall, these connectivity patterns suggest distinct functional roles, with Group 2 potentially acting as a hub for electrical coupling and facilitating chemical interactions with Group 3. Meanwhile, Group 3 also mediates intra‐group inhibition. These dynamics likely contribute to the modulation of cortical activity. Future studies with larger sample sizes for Group 1 cells (SBC‐like cells) are necessary to fully elucidate their connectivity patterns and functional significance.

### 3.4 L1INs Provide Broad GABAergic Inhibition to Deeper Layer Neurons in the mPFC

**FIGURE 5:**
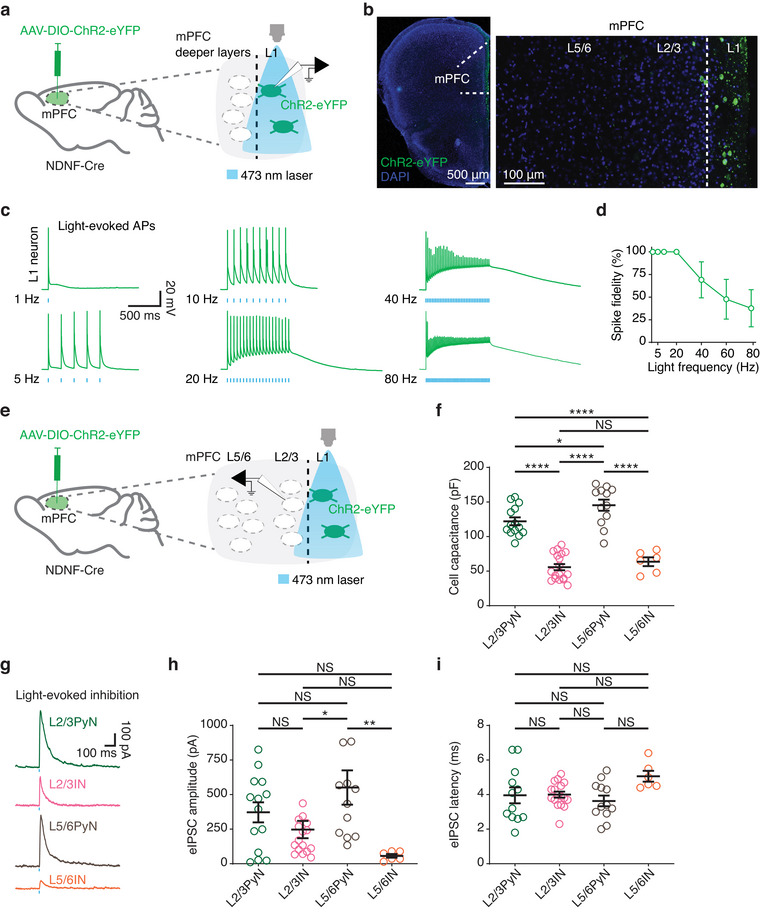
**Inhibitory control from mPFC NDNF^+^ L1INs to deeper layer neurons**. (a) Schematic illustrating intracerebral injections (left) and subsequent whole‐cell patch‐clamp recordings (right). (b) Representative image of a coronal slice of the mPFC, showing NDNF^+^ ChR2‐expressing cells localized in Layer 1. (c) Representative traces of light‐evoked action potentials from an NDNF^+^ L1IN at various stimulation frequencies (473 nm, 1 Hz, 5 Hz, 10 Hz, 20 Hz, 40 Hz, and 80 Hz, 5 ms duration with a 1‐s period). (d) Spike fidelity of NDNF^+^ L1INs in response to different stimulation frequencies, showing decreased fidelity beyond 20 Hz (*n* = 7 cells, seven slices from three mice; mixed‐effects model, F(6, 28) = 6.941, ***p = 0.0001). (e) Schematic illustrating whole‐cell recordings from deeper layer neurons in the mPFC during photoexcitation of NDNF^+^ L1INs. (f) Summary data of the cell capacitance of recorded deeper layer neurons (L2/3PyN, *n* = 14 cells; L2/3IN, *n* = 18 cells; L5/6PyN, *n* = 12 cells; L5/6IN, *n* = 6 cells; one‐way ANOVA, F(3, 46) = 51.95, ****p < 0.0001, *η* ^2^ = 0.7721; Tukey's post hoc test, ****p_(L2/3PyN vs. L2/3IN)_ < 0.0001, *p_(L2/3PyN vs. L5/6PyN)_ = 0.0437, ****p_(L2/3PyN vs. L5/6IN)_ < 0.0001, ****p_(L2/3IN vs. L5/6PyN)_ < 0.0001, *p*_(L2/3IN vs. L5/6IN)_ = 0.8704, ****p_(L5/6PyN vs. L5/6IN)_ < 0.0001). (g) Representative light‐evoked IPSC traces from pyramidal neurons and interneurons in various mPFC layers, triggered by photoexcitation of NDNF^+^ L1INs. (h) Summary of light‐evoked IPSC amplitude (L2/3PyN, *n* = 14 cells; L2/3IN, *n* = 18 cells; L5/6PyN, *n* = 12 cells; L5/6IN, *n* = 6 cells; one‐way ANOVA, F(3, 46) = 4.587, **p = 0.0068, *η* ^2^ = 0.2302; Tukey's post hoc test, *p*_(L2/3PyN vs. L2/3IN)_ = 0.5671, *p*_(L2/3PyN vs. L5/6PyN)_ = 0.4295, *p*_(L2/3PyN vs. L5/6IN)_ = 0.1498, *p_(L2/3IN vs. L5/6PyN)_ = 0.0314, *p*_(L2/3IN vs. L5/6IN)_ = 0.5970, **p_(L5/6PyN vs. L5/6IN)_ = 0.0095). (i) Summary of light‐evoked IPSC latency (L2/3PyN, *n* = 12 cells; L2/3IN, *n* = 17 cells; L5/6PyN, *n* = 12 cells; L5/6IN, *n* = 6 cells; one‐way ANOVA, F(3, 43) = 2.331, *p* = 0.0876, *η* ^2^ = 0.1399; Tukey's post hoc test, *p*_(L2/3PyN vs. L2/3IN)_ = 0.9996, *p*_(L2/3PyN vs. L5/6PyN)_ = 0.8862, *p*_(L2/3PyN vs. L5/6IN)_ = 0.1963, *p*_(L2/3IN vs. L5/6PyN)_ = 0.8118, *p*_(L2/3IN vs. L5/6IN)_ = 0.1868, *p*_(L5/6PyN vs. L5/6IN)_ = 0.0573). *Note:* The bar graphs depict the mean ± SEM. (a) AAV‐DIO‐ChR2‐eYFP was injected into the mPFC of adult NDNF‐Cre mice to selectively express ChR2 in NDNF^+^ L1INs. Three weeks later, whole‐cell patch‐clamp recordings from eYFP‐positive cells in mPFC layer 1 were conducted to evaluate photoexcitation (473 nm laser) of NDNF^+^ L1INs. (b) The left panel shows the entire mPFC, with a zoomed‐in view of the marked area on the right, displaying detailed cellular expression. Scale bars, 500 µm (left) and 100 µm (right). (c) Optogenetic stimulations are indicated in blue beneath each voltage trace. Scale bars, 20 mV × 500 ms. (g) Scale bars, 100 pA × 100 ms.

To address the inhibitory output from L1INs to deeper layer neurons, we conducted optogenetic activation combined with whole‐cell recordings in acute brain slices to examine the inhibitory effects of mPFC L1INs on neurons in Layers 2–6. To specifically activate L1INs in the mPFC, we utilized neuron‐derived neurotrophic factor (NDNF)‐IRES‐Cre knock‐in mice (Jackson Laboratory, # 030757), which express Cre in over two‐thirds of L1INs in the neocortex (Schuman et al. 2019). Three weeks after injecting AAV‐EF1a‐DIO‐hChR2(H134R)‐EYFP (UNC Vector Core) into the mPFC of NDNF‐Cre mice, we recorded light‐evoked activation of NDNF^+^ L1 cells (Figure 5a). Confocal microscopy confirmed that ChR2‐expressing cells were predominantly localized within L1 of the mPFC (Figure 5b), verifying that NDNF serves as a genetic marker largely restricted to L1 in adult mice.

To validate the optogenetic activation of L1INs, we measured light‐evoked (5 ms, 473 nm) responses of mPFC L1INs using whole‐cell patch‐clamp recordings (Figure 5a–d). All recorded L1INs (seven out of seven cells) reliably initiated action potentials in response to light stimulation. Previous studies have shown that the physiological firing rate of neocortical L1INs in vivo ranges from 1 to 12 Hz (Fan et al. 2020). To assess how fast L1INs can fire action potentials, we tested their spike fidelity in vitro brain slices using various stimulation frequencies. We found that spike fidelity decreased as the stimulation frequency exceeded 20 Hz (Figure 5c,d). The reduced spike fidelity at 40 Hz stimulation could be attributed to a depolarization block, as the membrane voltage accumulates during repeated stimulation (Figure 5c). For stimulation frequencies above 40 Hz, the reduced spike fidelity might result from the off‐kinetics limitation of ChR2(H134R) (Yizhar et al. 2011).

To identify the downstream targets of NDNF^+^ L1INs, we recorded light‐evoked postsynaptic responses from deeper layer neurons in the mPFC (Figure 5e–i). PyNs were distinguished from INs based on their characteristic pyramidal‐shaped soma and larger cell capacitance (Figure 5f), with layer identity determined by the location of their cell body. We found that the majority of recorded deeper layer neurons exhibited evoked inhibitory postsynaptic currents (eIPSCs, *n* = 47 out of 50 cells, 94.0%) (Figure 5g–i), indicating widespread inhibitory effects from NDNF^+^ L1INs, consistent with previous reports (Hartung et al. 2024). Among the recorded neuron subtypes, we observed that L5/6INs received the least inhibition from L1INs (eIPSC: L2/3PyN = 371.9 ± 72.3 pA; L2/3IN = 233.5 ± 60.3 pA; L5/6PyN = 551.5 ± 124.1 pA; L5/6IN = 57.0 ± 13.2 pA; one‐way ANOVA, F(3, 46) = 4.587, **p = 0.0068, *η* ^2^ = 0.2302) (Figure 5g,h), suggesting that L5/6INs are under the weakest inhibitory control from NDNF^+^ L1INs. Additionally, both L2/3 and L5/6 PyNs displayed larger variability in eIPSC amplitudes, implying that NDNF^+^ L1INs may produce differential inhibition across various PyN subtypes.

**FIGURE 6:**
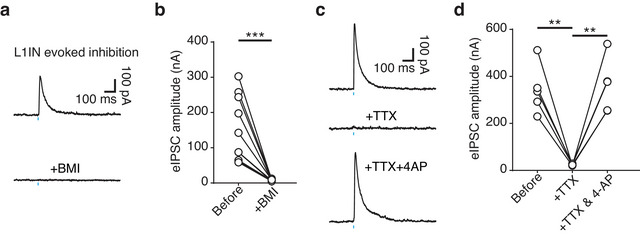
**mPFC NDNF^+^ L1INs provide monosynaptic GABAergic inhibition to deeper layer neurons**. (a) Light‐evoked IPSC traces from a deeper layer neuron before (top trace) and after (bottom trace) bath application of bicuculline (BMI, 10 µM). (b) BMI effectively blocked light‐evoked IPSCs in mPFC deeper layer neurons (*n* = 10 cells; paired *t*‐test, ***p = 0.0008, Cohen's *d* = 1.4703). (c) Representative traces showing the effect of TTX and 4‐AP on light‐evoked IPSCs. (d) Quantification of the effects of TTX and 4‐AP on the amplitude of light‐evoked IPSCs in mPFC deeper layer neurons corresponding to (c) (*n* = 5 cells; paired *t*‐test, **p_(before vs. TTX)_ = 0.0024, Cohen's *d* = 1.7533; paired *t*‐test, **p_(TTX vs. 4‐AP)_ = 0.0029, Cohen's *d* = 1.7382). *Note:* (a) The top trace shows an IPSC evoked by L1IN stimulation, which is absent in the bottom trace following BMI application, indicating GABAergic inhibition. Scale bars, 100 pA × 100 ms. (c) The first trace shows a light‐evoked IPSC, which is completely blocked by TTX (1 µM) in the second trace. After adding 4‐AP (200 µM) along with TTX, the third trace shows the restoration of the IPSC. Scale bars, 100 pA × 100 ms.

The time delay between the light pulse onset and the eIPSC response was approximately 4 ms across all neuron subtypes (L2/3PyN = 4.0 ± 0.5 ms; L2/3IN = 4.0 ± 0.2 ms; L5/6PyN = 3.6 ± 0.3 ms; L5/6IN = 5.1 ± 0.3 ms; one‐way ANOVA, F(3, 43) = 2.331, *p* = 0.0876, *η* ^2^ = 0.1399) (Figure 5i), corresponding to the time required for typical monosynaptic transmission. To confirm whether these inhibitory effects were mediated by monosynaptic GABAergic transmission, we applied several pharmacological approaches. eIPSCs were fully blocked by the bath application of BMI (10 µM), confirming GABAergic inhibition (before = 150.5 ± 29.3 pA; +BMI = 7.5 ± 0.7 pA; paired *t*‐test, ***p = 0.0008, and an effect size of Cohen's *d* = 1.4703) (Figure 6a,b). To verify monosynaptic transmission, we sequentially applied tetrodotoxin (TTX, 1 µM) and 4‐aminopyridine (4‐AP, 200 µM) into the bath solution (Linders et al. 2022) (Figure 6c,d). TTX blocked action potential‐dependent synaptic activity, thereby eliminating eIPSCs (before = 344.3 ± 46.9 pA; +TTX = 23.3 ± 1.6 pA; paired *t*‐test, **p = 0.0024, Cohen's *d* = 1.7533). The subsequent addition of 4‐AP, which blocks K^+^ channel‐mediated shunting of the light‐evoked response, restored the eIPSCs without action potential propagation (+TTX = 23.3 ± 1.6 pA; +TTX & 4‐AP = 360.9 ± 52.2 pA; paired *t*‐test, **p = 0.0029, Cohen's *d* = 1.7382). These results demonstrate that mPFC L1INs directly release GABA onto deeper layer neurons, producing inhibitory effects.

## 4 Discussions

The most common genetic markers for cortical INs include parvalbumin (PV), somatostatin (SST), and vasoactive intestinal peptide (VIP) (Rudy et al. 2011; Taniguchi et al. 2011; Wonders and Anderson 2006; Xu et al. 2010). However, PV‐expressing INs are absent in L1; SST‐ and VIP‐expressing INs together account for less than 10% of total L1INs (Almási et al. 2019; Xu et al. 2010). The lack of a specific genetic marker for L1INs makes it difficult to fully understand their functions. In 2011, NDNF, originally identified as A930038C07Rik, was recognized as a marker for cortical L1 cells through histological studies (Boyle et al. 2011). Subsequent single‐cell RNA sequencing studies confirmed that NDNF is highly expressed in NGCs (Tasic et al. 2016). More recently, NDNF‐Cre has been shown to be specifically expressed in approximately 70% of cortical L1INs (Schuman et al. 2019). Studies utilizing genetic tools to label NDNF‐expressing L1INs have since provided new insights into their properties and functions.

In this study, we investigated several aspects of L1INs in the mouse mPFC, including their morphological types, electrophysiological properties, interconnectivity, and inhibitory effects. We identified three morphologically distinct subtypes of L1INs and analyzed how these structural differences correspond to their electrophysiological properties, suggesting that morphological variation may contribute to the functional diversity of L1INs. Furthermore, we examined the interconnections between L1INs and created connectivity maps that describe their chemical and electrical connections across different groups. Lastly, we assessed the inhibitory effects of L1INs on deeper layer neurons and observed significant variability in the strength of inhibition among PyNs within the same layers, as well as differential inhibition of INs across different layers. These findings indicate that L1INs inhibit deep‐layer PyNs with substantial variability, suggesting that they may play a role in fine‐tuning neural circuits in the mPFC.

### 4.1 Morphological Types of L1INs Across Cortical Areas

The morphologies of L1INs in the rodent neocortex can be categorized into three main types based on their axonal extensions (Jiang et al. 2015; Jiang et al. 2013): NGCs, characterized by axonal arborizations confined to L1, and non‐NGCs, referred to as SBC‐like cells (SBC‐like), whose axons mostly arborize within L1 but send one or two side branches into deeper layers. The third subtype, eNGCs, is distinguished from classic NGCs by their extended axonal arborizations. These classification criteria have been applied to various cortical areas, including the primary visual cortex (V1) (Jiang et al. 2015), S1 (Schuman et al. 2019), and MEC (Shi et al. 2023) (Table 1). The tangential and vertical extensions of eNGCs and SBC‐like cells likely reflect distinct inhibitory strategies. For instance, eNGCs, with their elongated axonal arborizations, could provide dendritic inhibition to the apical dendrites of deeper layer PyNs, refining cortical processing (Abs et al. 2018). In contrast, SBC‐like cells, with their axonal branches reaching deeper layers, could target the somata of L2/3 INs, modulating cortical activity through disinhibition circuits (Letzkus et al. 2011). In our study, we observed these three morphological types of L1INs in the mPFC (Figure 2c), suggesting a degree of conservation in L1IN subtypes across cortical regions.

Both V1 and S1 contain eNGCs and SBC‐like cells, but their proportions differ: V1 has a higher percentage of SBC‐like cells (66%) compared with eNGCs (34%) (Jiang et al. 2015), whereas S1 is dominated by eNGCs (70%), with SBC‐like cells making up only 30% of the population (Schuman et al. 2019) (Table 1). In contrast, both the mPFC and MEC contain NGCs, which are characterized by shorter axonal arborizations compared with eNGCs. In the mPFC, NGCs account for 41.0% of the L1IN population (Figure 2g), whereas in the MEC, they represent an even larger proportion (57.7%) (Shi et al. 2023) (Table 1). However, NGCs were not characterized separately in V1 and S1, as they were not distinguished from eNGCs in the corresponding studies. As noted by Schuman et al. (2019), in S1, both NGCs and canopy cells were described as having elongated axonal arbors restricted to L1, though the dendritic extent of canopy cells was significantly larger than that of NGCs.

These observations highlight the significant variability in the proportions of L1IN morphological types across different cortical areas. V1 has the highest percentage of SBC‐like cells, whereas the MEC contains the lowest proportion. Additionally, the location of these cells within L1 varies by region. In both S1 and MEC, SBC‐like cells are typically located in the lower half of L1 (Schuman et al. 2019; Shi et al. 2023), a feature not seen in the mPFC (Figure 2f). These regional differences likely reflect distinct functional adaptations, suggesting that L1INs may not have uniform roles across cortical areas. Thus, region‐specific characterizations of L1IN functions are essential to accurately understand their roles.

### 4.2 Firing Patterns of L1INs Across Cortical Areas

L1INs display distinct firing patterns across different cortical regions (Table 2). In S1, the proportion of firing patterns changes with age. In juvenile mice (younger than P25), most L1INs exhibit LS firing patterns (76.4% in [Ma et al. 2014] and 79.7% in [Yao et al. 2016]), whereas the rest show burst spiking (BS) firing patterns (23.6% in [Ma et al. 2014] and 20.3% in [Yao et al. 2016]). However, in adult mice (older than 1 month), LS firing patterns are mostly observed in NDNF‐ and neuropeptide Y (NPY)‐positive L1INs, accounting for approximately 37.4% of the entire L1IN population. Meanwhile, the majority of NPY‐negative L1INs exhibit NLS firing patterns, comprising about 62.6% of the population (Schuman et al. 2019) (Table 2). These findings suggest that firing patterns in L1INs are not fixed throughout the lifespan and may evolve with age.

In V1, the majority of eNGCs (90.0%) exhibit LS firing patterns, with a smaller subset (10.0%) showing NLS patterns (Jiang et al. 2015). In contrast, SBC‐like cells show more variation, with 42.6% exhibiting BS firing patterns, 54.2% showing no burst NLS patterns, and only 3.2% displaying LS patterns. This differs from the MEC, where the majority of eNGCs (71.1%) display NLS patterns, whereas most NGCs (97.0%) and SBC‐like cells (75.9%) exhibit LS patterns (Shi et al. 2023) (Table 2). These regional differences indicate that firing patterns alone may not reliably predict L1IN morphology, suggesting that mathematical models for estimating L1IN morphology derived from one cortical region may not apply to other regions.

In the mPFC, the most common firing patterns observed were LS (64.4%) and NLS (35.6%) (Table 2). Among the 178 recorded L1INs, 2 neurons exhibited BS firing patterns (1.1%), which aligns with the ratio reported in a recent study on L1INs in the auditory cortex (Hartung et al. 2024). However, due to the small sample size, BS‐firing neurons were excluded from further analysis. Correlating firing patterns with morphology, we found that 60.0% of SBC‐like cells exhibited NLS patterns, whereas only 9.1% of NGCs and 25.0% of eNGCs displayed NLS patterns (Figure 1b). When comparing across regions, SBC‐like cells in both the mPFC and V1 display a higher proportion of NLS firing patterns (60% in mPFC; 54.2% in V1), though their relative populations differ, with SBC‐like cells accounting for 30.8% in the mPFC and 66% in V1 (Table 1). On the other hand, the proportion of eNGCs in the MEC (25.7%) is comparable to that in the mPFC (28.2%) (Table 1), but most eNGCs in MEC exhibit NLS patterns (71.1%), whereas only 25% of eNGCs in the mPFC display NLS patterns. These findings further emphasize that firing patterns alone cannot reliably predict the morphology of L1INs across different cortical areas.

The phenomenon of LS and NLS firing patterns has been observed in other brain regions, such as the dorsal cochlear nucleus (DCN) of the guinea pig (Manis 1990), where the latency to the first spike can be influenced by hyperpolarizing current before depolarization. This concept has been further validated through mathematical models (Kanold and Manis 2001; Meng et al. 2011). It has been suggested that transient K^+^‐conductance may control the firing patterns of mouse NGCs (Chittajallu et al. 2020). Given the variation in firing patterns among morphologically similar neuron types across different brain regions, it is essential to determine whether these patterns are governed by common or distinct mechanisms. More importantly, the physiological significance of having different firing patterns remains to be explored. Addressing these questions could significantly bridge the gap between in vitro data and in vivo studies.

### 4.3 Interconnectivity Between L1INs

Neocortical L1INs communicate with each other through both chemical and electrical connections (Chu et al. 2003; Fan et al. 2020; Merriam et al. 2005; Yao et al. 2016). Chemical inhibition between L1INs, known as lateral inhibition, has been observed in the somatosensory cortex during whisker stimulation and is thought to contribute to the precise timing of sensory‐evoked responses (Fan et al. 2020). Electrical coupling between L1INs, described in the developing somatosensory cortex, is believed to promote the formation of bidirectional rather than unidirectional chemical connections (Yao et al. 2016). This positive correlation between electrical coupling and bidirectional chemical connections has been shown in L1INs of the MEC (Shi et al. 2023). Thus, mutual chemical inhibition may facilitate synchrony between L1INs, as hypothesized in earlier studies (Hu et al. 2011; Van Vreeswijk et al. 1994). From this perspective, the combination of bidirectional chemical connections and electrical coupling may enhance L1IN synchronization, although the physiological conditions under which this synchronization occurs remain unclear.

In contrast, a recent in vivo study of L1INs in the mouse V1 revealed a diverse range of sensory and motor responses within the L1IN population, with these responses appearing randomly distributed (Lukas et al. 2019). Given the sharp decline in connection ratios between L1INs as distance increases, the importance of synchronizing nearby L1INs remains to be clarified.

Our findings revealed the presence of both electrical and chemical connections between L1INs in the mPFC, likely reflecting a dual mechanism for regulating circuit dynamics. Electrical coupling may facilitate synchronization to maintain network coherence during cognitive tasks (Bennett and Zukin 2004; Draguhn et al. 1998), whereas GABAergic inhibition could refine circuit outputs under specific conditions (Cohen‐Kashi Malina et al. 2021; Letzkus et al. 2011; Tremblay et al. 2016).

The functional importance of these electrical and chemical connections between L1INs in the mPFC remains an open question. Future research should investigate how these connectivity patterns are modulated during behavioral tasks or under pathological conditions, which could offer deeper insights into the role of L1INs in cortical function.

### 4.4 Inhibitory Effects of L1INs on Deeper Layer Neurons

The inhibitory output from L1INs to deeper layer neurons plays a critical role in gating information flow and modulating neuronal activity in several cortical areas, including the auditory cortex (Abs et al. 2018; Hartung et al. 2024; Letzkus et al. 2011; Merriam et al. 2005), sensorimotor cortex (Jiang et al. 2013), somatosensory cortex (Muralidhar et al. 2013; Schuman et al. 2019; Yao et al. 2016), visual cortex (Cohen‐Kashi Malina et al. 2021; Fan et al. 2020; Ibrahim et al. 2021; Jiang et al. 2015), prefrontal cortex (Anastasiades et al. 2021; Cruikshank et al. 2012), and MEC (Shi et al. 2023).

From a molecular identity perspective, NDNF^+^ L1INs preferentially inhibit L2/3 PyNs, L2/3 PV^+^ INs, and L2/3 VIP^+^ INs, but not L2/3 SST^+^ INs (Abs et al. 2018; Anastasiades et al. 2021; Cohen‐Kashi Malina et al. 2021; Hartung et al. 2024). These findings provide a foundation for understanding L1INs’ involvement in both inhibitory and disinhibitory circuits. For instance, dendritic inhibition by L1INs has been shown to regulate learning‐related responses in the auditory cortex (Abs et al. 2018), whereas L1IN‐mediated disinhibition plays a critical role in fear learning (Letzkus et al. 2011). However, the specific molecular subtypes of L1INs that govern these pathways remain unclear (Hartung et al. 2024).

From a morphological perspective, it has been suggested that SBC‐like cells preferentially target L2/3 INs but not L2/3 PyNs, potentially contributing to disinhibitory circuits. In contrast, eNGCs inhibit both L2/3 INs and L2/3 PyNs, allowing them to participate in both inhibitory and disinhibitory mechanisms (Jiang et al. 2015; Shi et al. 2023). Interestingly, NDNF^+^ L1INs inhibit the majority of L2/3 PyNs (90.9% in the auditory cortex [Hartung et al. 2024]; 85.7% in the mPFC from our data), but this inhibitory ratio drops significantly when analyzed using multiple whole‐cell recordings (8.8% in the MEC [Shi et al. 2023]; 15.9% in S1 [Jiang et al. 2015]).

To explain this inconsistency, we propose a differential inhibition hypothesis, which suggests that subsets of L1INs inhibit L2/3 PyNs according to specific rules. As a result, only a small proportion of PyNs are inhibited by individual L1INs. But when all subsets of L1INs are activated simultaneously (e.g., through optogenetic methods), nearly all L2/3 PyNs receive inhibition from L1INs. If so, the exact rules by which L1INs differentially inhibit deeper layer neurons remain to be elucidated. Additionally, our findings revealed that L1IN inhibition extends beyond L2/3 and to L5/6, though L5/6 INs receive minimal inhibition from L1INs. This could be attributed to L5/6 INs typically not extending their dendrites into superficial layers. However, we did not distinguish these downstream neurons using specific molecular markers. A more comprehensive characterization of the PyN subtypes receiving differential inhibition from distinct L1IN subsets could provide valuable insights into the functional organization of these circuits.

## Author Contributions

Conceptualization: Chen Shen and Lin Mei. Methodology: Chen Shen, Wen‐Cheng Xiong, and Lin Mei. Investigation: Chen Shen and Wanpeng Cui. Data analysis: Chen Shen. Writing: Chen Shen, Wen‐Cheng Xiong, and Lin Mei. Funding acquisition: Lin Mei. Supervision: Wanpeng Cui, Wen‐Cheng Xiong, and Lin Mei.

## Conflicts of Interest

The authors declare no conflicts of interest.

### Peer Review

The peer review history for this article is available at https://publons.com/publon/10.1002/cne.70030.

## Supporting information



Figure S1 Mapping and distribution of reconstructed mPFC L1INs across coronal sections.

Figure S2 Validity check for hierarchical clustering of morphological–electrophysiological relationships.

Table S1 Summary of statistical tests and effect sizes used in this study.

## Data Availability

Requests for further information and resources should be directed to the lead contact, L.M. (linmei@cimrbj.ac.cn).
