# Supplementary material for: Heterogeneity of Layer 1 Interneurons in the Mouse Medial Prefrontal Cortex
Source: J Comp Neurol. 2025 Mar 4;533(3):e70030. doi: 10.1002/cne.70030 (PMC11877257; doi:10.1002/cne.70030)
Supplement: Supplementary file 1 — Figure S1 Mapping and distribution of reconstructed mPFC L1INs across coronal sections. [file CNE-533-e70030-s001.pdf]

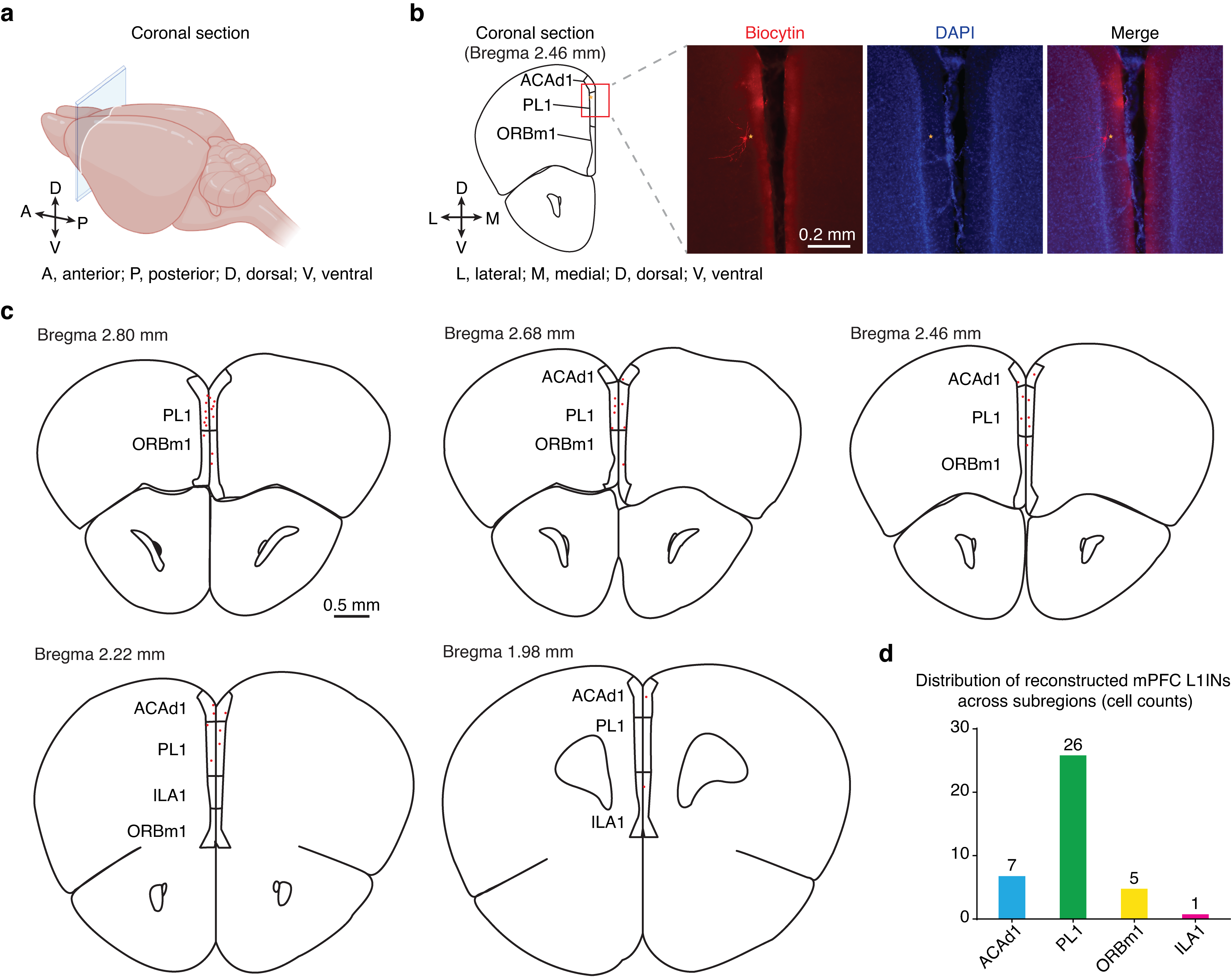
**Figure S1** | **Mapping and distribution of reconstructed mPFC L1INs across coronal sections.**

(**a**) Illustration shows the coronal sections of mouse mPFC were prepared for whole-cell patch-clamp recording and subsequent morphological reconstruction.

(**b**) Coronal section at Bregma 2.46 mm, illustrating a representative L1IN (star) that was recorded and biocytin-filled (red). Scale bar, 0.2 mm.

(**c**) Approximate locations of reconstructed L1INs (red dots) mapped on a standard mouse mPFC atlas at various Bregma levels (2.80 mm to 1.98 mm), adapted from (Bakker et al., 2015; Lein et al., 2007).

(**d**) Bar graph showing the number of reconstructed L1INs located in four mPFC subregions: ACAd1, PL1, ORBm1, and ILA1.

Abbreviations: ACAd1: Anterior cingulate area, dorsal part, layer 1; PL1: Prelimbic area, layer 1; ORBm1: Orbital area, medial part, layer 1; ILA1: Infralimbic area, layer 1.
