# Supplementary material for: Heterogeneity of Layer 1 Interneurons in the Mouse Medial Prefrontal Cortex
Source: J Comp Neurol. 2025 Mar 4;533(3):e70030. doi: 10.1002/cne.70030 (PMC11877257; doi:10.1002/cne.70030)
Supplement: Supplementary file 2 — Figure S2 Validity check for hierarchical clustering of morphological–electrophysiological relationships. [file CNE-533-e70030-s003.pdf]

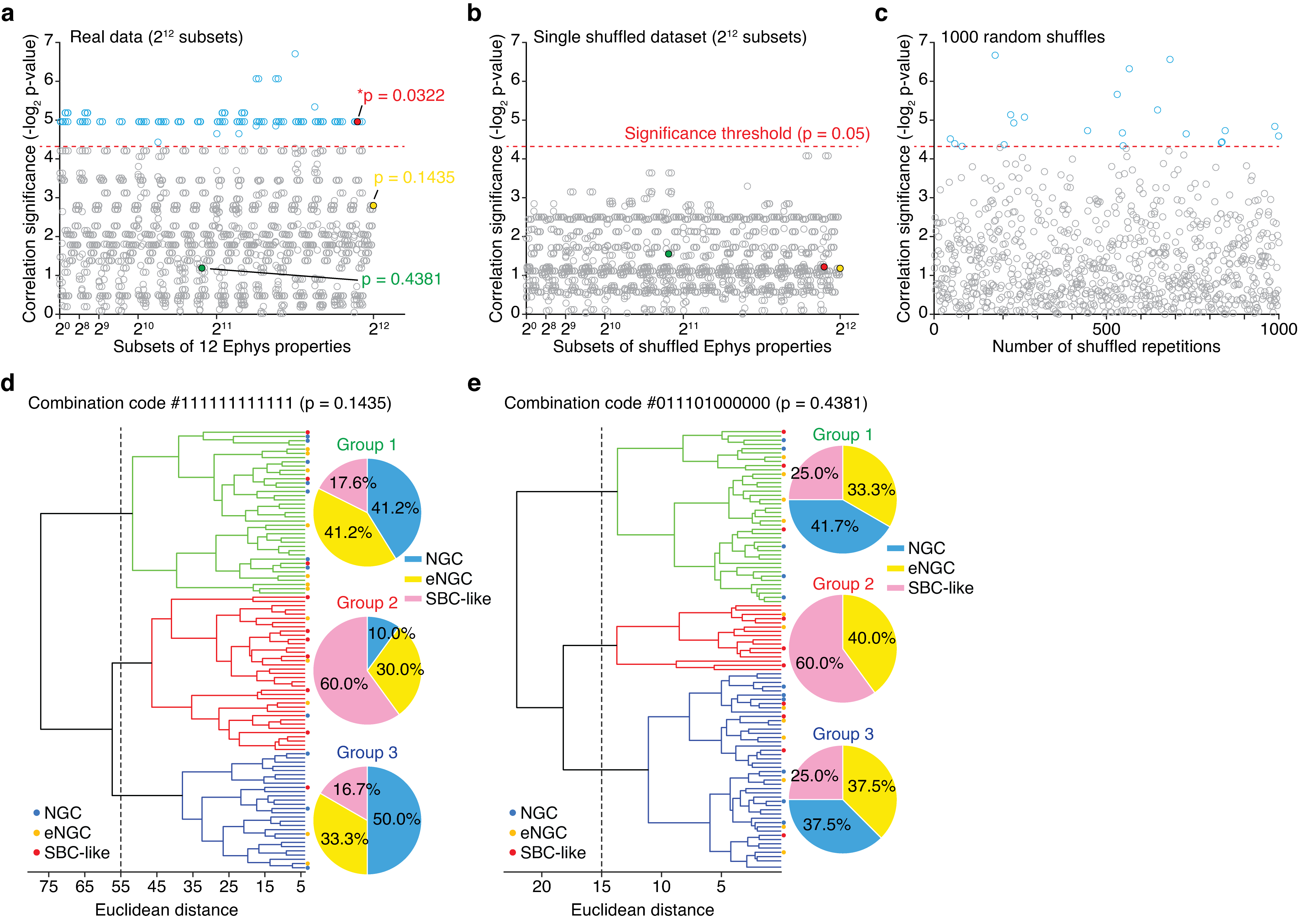
 **Figure S2** | **Validity check for hierarchical clustering of morphological–electrophysiological relationships.**

(**a**) Correlation between morphological types and electrophysiological groupings. Each circle represents the significance (−log_2_ p-value) of a chi-squared test relating morphological types to one subset of electrophysiological properties. The dashed red line (p = 0.05) marks the significance threshold. A total of 4,095 subsets were tested, of which 287 surpassed this threshold. The red dot corresponds to the nine-parameter subset used in Figure 1d. The yellow dot includes all 12 parameters (see Supplementary Figure 2d). The green dot is the subset containing only action potential waveform measures (threshold, peak amplitude, spike half-width, and maximum spike slope; see Supplementary Figure 2e).

(**b**) The same chi-squared correlation analysis as in (**a**), but each electrophysiological parameter was randomly shuffled among cells. Of the 4,095 subsets tested, none exceeded the significance threshold. The colored dots correspond to the same subsets highlighted in (**a**). Notably, none of these colored subsets in the shuffled data exceeded the significance threshold.

(**c**) To verify the robustness of the nine-parameter subset from Figure 1d, we shuffled each electrophysiological parameter among cells 1,000 times and tested each shuffle for correlation with morphological types. Only 21 out of 1,000 (≈2.1%) met the p < 0.05 criterion, which is below the 5% level.

(**d**) Dendrogram of all 12 electrophysiological parameters. A hierarchical cluster tree was constructed using Ward’s method (see Materials and Methods 2.4), revealing three distinct electrophysiological cell groups. Colored dots between the dendrogram and the pie charts indicate morphological identity (blue: NGC, yellow: eNGC, red: SBC-like). The pie charts show the proportions of morphological types within each electrophysiological groups (Group 1, NGC = 7, eNGC = 7, SBC-like = 3; Group 2, NGC = 1, eNGC = 3, SBC-like = 6; Group 3, NGC = 3, eNGC = 2, SBC-like = 1). A chi-squared test (χ²(df = 4) = 6.859, p = 0.1435, Cohen’s w = 0.4559) found no significant morphological distribution differences among these groups.

(**e**) Same as (**d**) but using only action potential waveform measures—threshold, peak amplitude, spike half-width, and maximum spike slope—to form three electrophysiological groups (Group 1, NGC = 5, eNGC = 4, SBC-like = 3; Group 2, NGC = 0, eNGC = 2, SBC-like = 3; Group 3, NGC = 6, eNGC = 6, SBC-like = 4; χ²(df = 4) = 3.769, p = 0.4381, Cohen’s w = 0.3379).
