# Supplementary material for: Heterogeneity of Layer 1 Interneurons in the Mouse Medial Prefrontal Cortex
Source: J Comp Neurol. 2025 Mar 4;533(3):e70030. doi: 10.1002/cne.70030 (PMC11877257; doi:10.1002/cne.70030)
Supplement: Supplementary file 3 — Table S1 Summary of statistical tests and effect sizes used in this study. [file CNE-533-e70030-s002.docx]

**Table S1.** **Summary of statistical tests and effect sizes** **used in this study.**

| Figure # | Panel # | Description | Tests | p-values | Effect sizes | |
| --- | --- | --- | --- | --- | --- | --- |
| Figure 1 | b | Different distributions of firing patterns across morphological types | chi-squared | 0.0355* | Cohen’s w = | 0.4499 |
|  | d | Different distributions of morphologies across electrophysiological groups | chi-squared | 0.0322* | Cohen’s w = | 0.5654 |
| Figure 2 | e | Axonal tangential extents among NGC, eNGC and SBC-like cells | one-way ANOVA | < 0.0001**** | eta squared = | 0.5435 |
|  | e | Axonal vertical extents among NGC, eNGC and SBC-like cells | one-way ANOVA | < 0.0001**** | eta squared = | 0.6971 |
|  | f | Soma depth among NGC, eNGC and SBC-like cells | one-way ANOVA | 0.5345 | eta squared = | 0.0342 |
|  | g | Neuron population among groups match evenly distributed expectation | chi-squared | 0.7722 | Cohen’s w = | 0.1151 |
| Figure 3 | f | Electrical coupling strength in either direction | paired t-test | 0.5587 | Cohen’s d = | 0.1612 |
| Figure 4 | d | Response latency between chemical and electrical connections | unpaired t-test | < 0.0001**** | Cohen’s d = | 1.7258 |
| Figure 5 | f | Cell capacitance among cell types | one-way ANOVA | < 0.0001**** | eta squared = | 0.7721 |
|  | h | eIPSC amplitude among cell types | one-way ANOVA | 0.0068** | eta squared = | 0.2302 |
|  | i | eIPSC latency among cell types | one-way ANOVA | 0.0876 | eta squared = | 0.1399 |
| Figure 6 | b | BMI effects on eIPSC amplitude | paired t-test | 0.0008*** | Cohen’s d = | 1.4703 |
|  | d (before vs. TTX) | TTX effects on eIPSC amplitude | paired t-test | 0.0024 | Cohen’s d = | 1.7533 |
|  | d (TTX vs. 4-AP) | TTX&4-AP effects on eIPSC amplitude | paired t-test | 0.0029 | Cohen’s d = | 1.7382 |
| Figure S2 | d | Different distributions of morphologies across electrophysiological groups | chi-squared | 0.1435 | Cohen’s w = | 0.4559 |
|  | e | Different distributions of morphologies across electrophysiological groups | chi-squared | 0.4381 | Cohen’s w = | 0.3379 |
| Table 1 |  | Morphologies across brain areas | chi-squared | <0.0001**** | Cohen’s w = | 0.7266 |
| Table 2 |  | Firing patterns across brain areas | chi-squared | <0.0001**** | Cohen’s w = | 0.6241 |
| Table 3 |  | Membrane potential across morphological types | one-way ANOVA | 0.2706 | eta squared = | 0.0834 |
|  |  | Threshold across morphological types |  | 0.2623 | eta squared = | 0.0854 |
|  |  | Peak amplitude across morphological types |  | 0.5492 | eta squared = | 0.0392 |
|  |  | Halfwidth across morphological types |  | 0.9683 | eta squared = | 0.0021 |
|  |  | First spike latency across morphological types |  | 0.1307 | eta squared = | 0.1269 |
|  |  | Spike max rise slope across morphological types |  | 0.5121 | eta squared = | 0.0436 |
|  |  | AHP amplitude across morphological types |  | 0.3696 | eta squared = | 0.0642 |
|  |  | AHP duration across morphological types |  | 0.8489 | eta squared = | 0.0109 |
|  |  | Input resistance across morphological types |  | 0.021* | eta squared = | 0.2271 |
|  |  | Time constant across morphological types |  | 0.0961 | eta squared = | 0.1446 |
|  |  | Cell capacitance across morphological types |  | 0.3849 | eta squared = | 0.0617 |
|  |  | Depolarizing hump across morphological types |  | 0.5669 | eta squared = | 0.0371 |
| Table 4 |  | Membrane potential across Ephys groupings | one-way ANOVA | 0.0657 | eta squared = | 0.0525 |
|  |  | Threshold across Ephys groupings |  | 0.0075** | eta squared = | 0.0924 |
|  |  | Peak amplitude across Ephys groupings |  | 0.2728 | eta squared = | 0.0254 |
|  |  | Halfwidth across Ephys groupings |  | 0.9559 | eta squared = | 0.0009 |
|  |  | First spike latency across Ephys groupings |  | 0.3955 | eta squared = | 0.0182 |
|  |  | Spike max rise slope across Ephys groupings |  | 0.0835 | eta squared = | 0.0479 |
|  |  | AHP amplitude across Ephys groupings |  | 0.0871 | eta squared = | 0.0472 |
|  |  | AHP duration across Ephys groupings |  | 0.0296* | eta squared = | 0.0673 |
|  |  | Input resistance across Ephys groupings |  | <0.0001**** | eta squared = | 0.7891 |
|  |  | Time constant across Ephys groupings |  | <0.0001**** | eta squared = | 0.4649 |
|  |  | Cell capacitance across Ephys groupings |  | 0.0479* | eta squared = | 0.0584 |
|  |  | Depolarizing hump across Ephys groupings |  | 0.2021 | eta squared = | 0.0312 |
